# Anthropometric Development in Children: Possible Changes in Body Mass, Basal Metabolic Rate and Inflammatory Status

**DOI:** 10.3390/children8060455

**Published:** 2021-05-28

**Authors:** Roxana Maria Martin-Hadmaș, Ștefan Adrian Martin, Adela Romonți, Cristina Oana Mărginean

**Affiliations:** 1 Department of Community Nutrition and Food Safety, “George Emil Palade” University of Medicine, Pharmacy, Science and Technology from Târgu Mureș, Gheorghe Marinescu 38, 540139 Mureș, Romania; roxana.hadmas@umfst.ro (R.M.M.-H.); adela.romonti@umfst.ro (A.R.); 2 Center for Advanced Medical and Pharmaceutical Research, Department of Physiology, “George Emil Palade” University of Medicine, Pharmacy, Science and Technology from Târgu Mureș, Gheorghe Marinescu 38, 540139 Mureș, Romania; 3 Department of Pediatrics I, “George Emil Palade “University of Medicine, Pharmacy, Science and Technology from Târgu Mureș, Gheorghe Marinescu 38, 540139 Mureș, Romania; oana.marginean@umfst.ro

**Keywords:** interleukins, body mass, energy intake, children

## Abstract

(1) Background: Worldwide, public health policies focus on studying dietary patterns and the related anthropometric changes in children. Their aim is to improve the measures meant to reduce global malnutrition rates. Our goal was to study the main changes in the inflammatory status related to anthropometric changes and total daily energy intake. (2) Methods: We tested the study hypothesis by analyzing serum IL-6 and IL-8 levels, cholesterol and triglycerides values, as well as total proteins and creatinine levels, RMR, and food journals in a sample of 160 healthy subjects aged between 6 and 12 years old. (3) Results: IL-6 was correlated with the skinfold values. Changes in the skinfolds were significantly correlated with total proteins and triglycerides. Both weight for age and height for age were related to the skinfold values. Through the BMR measurements, peak carbohydrate metabolism changed significantly based upon IL-6 values, which were significantly correlated with the respiratory coefficient values. Based on the basal metabolic rate, an increased IL-8 ratio was related to the RQ value. (4) Conclusions: Skinfolds have been significantly correlated with IL-6 and IL-8 levels. With changes in body weight, we encountered differences in both serum cholesterol and serum triglycerides values, unlike total proteins and creatinine, which failed to change.

## 1. Introduction

Worldwide, public health policies focus on studying dietary patterns and related anthropometric changes in children [1,2]. The aim is to improve the measures meant to reduce global malnutrition rates. However, the methods implemented so far have generally been unsuccessful, because the rate of malnutrition is constantly increasing in young individuals.

By definition, childhood obesity refers to an excessive body weight, with an abnormal percentage of body fat. Based on the World Health Organization’s report for 2016, 18% of children aged between 5 and 19 years old were diagnosed with obesity, while in 2020, 38.9 million children aged under 5 years old were overweight and 45.4 million were malnourished. In cases of children aged under 5 years, in Europe, the obesity rate has increased from 3 million to 3.2 million during the last 20 years [3,4]. Therefore, the risk of insulin resistance, type two diabetes, hypertension, dyslipidemia, cardiovascular disease, and hyperuricemia will increase, affecting life quality and life expectancy [5].

Numerous studies have addressed relevant subjects regarding obesity [6,7,8,9,10]. Some of them have assessed the prevalence of obesity by using anthropometry development data [11,12,13], as well as information regarding daily food intake [14,15] and physical activity levels [16,17,18].

By following similar study methodologies, other authors [19,20] appreciated the population profile in order to determine the risk of obesity among both young and old-aged individuals. One study [21] offered important data regarding the psycho-nutritional approach, while other papers have published relevant data regarding the pathophysiological changes [22,23,24] in the human body. Many researchers [25,26,27] used initial screening tools to analyze changes in blood pressure, fat mass, and blood fat profile, along with inflammatory status by determining interleukin 6 (IL-6), interleukin 8 (IL-8), interleukin 3 (IL-3), interleukin 10 (IL-10) and interleukin 14 (IL-14) levels. Therefore, in adult participants, abnormal changes in blood pressure, body weight and blood fat profiles, specifically due to changes in cholesterol, triglycerides, low-density lipoproteins, high-density lipoproteins values, induced changes in inflammatory status [28,29,30].

Contemporary obesity-based research has taken on a much more practical approach. The metabolic syndrome was previously associated with changes in both daily energy needs—daily energy intake, daily level of physical activity, along with a higher-than-normal body weight [25,26,27,28,29]. This is why some authors approached obesity-related subjects by using the main pathological state [30,31,32]. In many cases, they seem to emphasize changes in interleukins, mostly related to the body weight and body mass distribution, especially in adult participants [33].

The number of papers that have studied changes in IL-6, IL-8, TNF-α, IL-10, IL-13, and IFN-γ status following abnormal body weight has increased [34,35,36,37]. Most of these studies were conducted on adult participants and tested the inflammatory status based on naturally occurring protein components that mediate cell communication, cell growth, and cell differentiation [37,38]. These proteins are known as interleukins and cytokines, which consist of multiple messenger molecules.

In several cases, the interleukins are linked to the level of immune cell maturation as well as the level of inflammation within the human body [39]. Obesity induces chronic low-grade inflammation which can increase the serum level of interleukins [33], of which the most frequently assessed are IL-6 and the IL-8. Furthermore, some authors state that in healthy individuals, IL-6 infusion can increase lipolysis and therefore energy requirements [40,41]. This is an important point, according to which individual responses can be different due to one of the three different action mechanisms: (a) proinflammatory; (b) anti-inflammatory; and (c) noninflammatory. Similar conclusions were drawn by using IL-8 plasma concentrations, which are high in obese individuals [41,42]. Moreover, IL-8 is related to the TNF-alpha system, which can increase energy expenditure and therefore total energy intake in a proinflammatory phase [43]. Such changes differ in overweight and obese individuals without other associated comorbidities. However, less information is available for young participants.

A small number of research papers studied the inflammatory status in normal weight, overweight or obese children [44,45]. Therefore, our goal was to assess the main changes in inflammatory status by measuring IL-6 and IL-8 status along with anthropometric development and total daily energy intake in order to identify possible systemic inflammatory damage in young individuals.

## 2. Materials and Methods

### 2.1. Study Design

We performed a prospective, observational, analytical study between November 2020 and March 2021 in the Advanced Medical and Pharmaceutical Research Center (CCAMF), George Emil Palade University of Medicine, Pharmacy, Science and Technology of Târgu Mureș, in a tertiary center in Romania, with 160 children. Eight participants were excluded from the study sample because they did not attend in accordance with the established research schedule.

All the steps of the study were explained to children and their parents/caregivers prior to their inclusion. Thus, the parents/caregivers signed the informed consent on behalf of their children, while all children gave their verbal assent prior to the inclusion in our study. Our study was approved by the Ethics Committee of the University of Medicine, Pharmacy, Sciences and Technology Târgu Mureș (No. 259/14.11.2018), and it was performed according to the principles of the Declaration of Helsinki.

### 2.2. Study Participants

To create the study sample, the individuals had to meet the following inclusion criteria: (1) children aged from 6 to 12 years old, and (2) no health problems, regardless of body weight. The following exclusion criteria were used: acute pathologies, including viral infections, which can influence the resting energy expenditure; chronic pathologies; congenital disorders; or food allergies.

### 2.3. Test Applied

#### 2.3.1. Basal Metabolic Rate Measurement

The basal metabolic rate (BMR) was measured once during the early morning, after an 8 h fasting period. We used the Cortex Metalyzer 3B device (Leipzig, Germany), which was calibrated with known O_2_ (16%) and CO_2_ (5%) concentrations before each test. The flow meter was also calibrated by simulating respiratory volumes that reached up to 0.3–0.6 L per respiratory cycle.

For each measurement, we manually defined the steady state point after 00:20:00 to 00:30:00 (hh:mm:ss) minutes of the testing period. To reach the steady state level, we pre-set an acceptable deviation of 10% in oxygen consumption (VO_2_), 6% in carbon dioxide elimination (VCO_2_), and 3% for the respiratory coefficient (RQ). Prior the test, each participant had an adaptation period of 10 min long in order to adapt to the testing environment. Oxygen saturation (O_2sat_, %), heart rate (HR, b/min), and both systolic (SBP, mmHg) and diastolic (DBP, mmHg) blood pressure were measured by using General Electric HEALTCARE B20 (Chicago, IL, USA) equipment, during the testing environment adaptation period.

During each test, we measured the BMR reported as kilocalories (kcal) per minute/hour/day. Carbohydrate metabolism (CHO, grams/day; %) and fat metabolism (Fat, grams/day; %) were also reported, along with oxygen consumption (VO_2_), carbon dioxide production (VCO_2_), and the respiratory coefficient (RQ). Furthermore, MetaSoft 3 software was used to calculate the theoretical resting energy expenditure (REE) by applying the Harris–Benedict equation [46].

#### 2.3.2. Anthropometric Measurements

Anthropometric measurements took place for each participant on the same day as the BMR measurement, after an 8 h fasting period. Each measurement took place in the presence of the parents/caregivers. All anthropometric measurements were conducted in underwear, without shoes or any other item of clothing that could have influenced the measurement result.

The weight (kg), the height (cm), and the skinfolds (mm) were all measured during one single visit. The weight was measured three times in a row, at 30 s intervals, by using the ADE GmbH M304040-01 calibrated scale (Germany) in an upright position. In similar conditions and with the same equipment, we measured the height in an upright position. For both anthropometric measurements, the accepted error was <1.5%.

Weight and age were measured to determine the weight for age and the height for age percentiles (%). In addition, the body mass index (BMI) was calculated and expressed as a percentile by referring to the BMI for age (%). The normal percentage range was appreciated as between 5 and 85%; <5% was underweight; >95% was obese [47].

The skin folds were measured by using the HARPENDEN Professional Skinfold (Southam, UK). The thickness of the biceps, the triceps, the subscapular, the Suprailiac site, the abdominal, and the thigh and the calf skinfolds were measured and reported as the sum of seven skinfolds (mm). By using the biceps, the triceps, the Suprailiac site and the abdominal skinfold, with reference to age and gender, we determined the body mass, muscle mass (%) and the fat mass (%) in the Durnin–Womersley equation [48].

### 2.4. Nutritional Analysis

We obtained the participants’ food intake reports by using a three-day (*n* = 3) period food journal. The parents/caregivers of the participants made the food journal available. The journal contained information such as the serving time (hour: minute), the quantity (grams/kilograms) and type of food consumed, which further gave us the possibility to calculate the participant’s energy intake (kcal/day) in relation to the measured BMR value. In this phase, we did not analyze the food intake from a qualitative point of view, but only from a quantitative and energetic approach, by using the USDA’s Food Composition Databases, property of the United States’ Department of Agriculture (2020) [49].

### 2.5. Blood Samples

Twelve milliliters of venous blood were extracted from each participant after an 8 h fasting period. Each blood sample was held at room temperature for 15 min, centrifuged at 80 rpm, and sampled in 12 µL micro tubes, stored, and frozen at −80 °C until analysis. Blood sample analysis was performed using a Cobas Integra 400 Plus system (Rotkreuz, Switzerland). The analysis included the total cholesterol (80–200 mg/dL), creatinine (<8 years old: 0.40–0.60 mg/dL; 8–10 years old: 0.39–0.73 mg/dL), triglycerides (50–150 mg/dL) and total proteins (66–87 g/L).

To assess the inflammatory status, we determined both IL-6 (<3.8 pg/mL) and IL-8 (<15 pg/mL) serum levels. Each parameter was measured from the sample by using the DYNEX DSX AUTOMATED ELISA SYSTEM (Cheshire, UK) based on an immuno-enzymatic assay.

The reagents were brought to room temperature. During the first stage, the samples, standards, and controls were added to the wells, which were lined with a monoclonal antibody for IL-6 and IL-8. After the incubation period, the IL-6 and IL-8 present in the sample, standard or control unrelated to the fixed antibody, was removed by washing with a wash buffer. After the washing process, a solution containing a monoclonal antibody to IL-6 labeled with an enzyme—biotin—was added. We further added the streptavidin–HRP conjugate. The absorbance of each well was read spectrophotometrically by the analyzer. The IL-6 and IL-8 concentrations were determined after reading the absorbances for each well and after the interpolation on the calibration curve. The cytokine concentration was directly proportional to the color intensity of the well. Following the measurement procedure, we had an intra- and interassay coefficients of less than 5.3% and less than 8.3%, respectively, for both IL-6 and IL-8.

### 2.6. Statistical Evaluation

The statistical evaluation was carried out with GraphPad Prism 6.0 software, with a level of significance set at α = 0.05. The tests used for inferential assessment were the Mann–Whitney test for differences between two items and the Spearman’s r test for assessing the relationship between two analyzed parameters. The data are presented by using descriptive data such as the median value, the minimum–maximum values, and the variation coefficient (CV). Due to food intake variability, average values were not used in this study.

## 3. Results

### 3.1. Demographic Analysis

The median age in the study group was 10 years old, with a minimum age of 7 years old and a maximum age of 12 years old. The median height was 158 cm, and the median body weight was 46.1 kg. The skinfold sum was 58.75 mm, with 16.7% median fat body mass, as further described in Table 1.

There was an acceptable variability in the body weight values (26.07%), with high variability over the sum of the skinfolds (46.16%), weight for age (53.78%), and height for age percentiles (55.19%). However, the skinfold sum was correlated significantly with all anthropometric results, including the BMI for age (*p* = 0.0001, r = 0.433), weight for age (*p* = 0.0001, r = 0.391), and height for age (*p* = 0.0069, r = 0.218) percentiles. Overall, with weight, blood pressure changed. DBP increased with body weight (*p* = 0.0001, r = 0.391), unlike SBP (*p* > 0.05), whereas the percentage of lean tissue dropped with the increasing skinfold values (*p* = 0.0001, r = −0.3736).

### 3.2. Basal Metabolic Rate Results

The basal metabolic rate reached a median value of 1676 kcal/day with a minimum value of 995 kcal/day and a maximum value of 2520 kcal/day. The energy requirement obtained by calculation was 1376 kcal/day, with a difference of 21.80% from the measured BMR value (*p* = 0.0001, r = 0.653). Furthermore, by applying a more practical analysis, we observed that the median daily energy intake reached 1854 kcal/day. This value was +10.2% above the measured BMR and +34.36% above the theoretical energy expenditure.

During the BMR test, we measured the median VO_2_ at 0.24 L/min and the median VCO_2_ at 0.21 L/min, with 0.81 RQ. Allover, the carbohydrate metabolism was between 50 and 279 g/day, with a 221 g/day median value, while the fat metabolism was between 57 and 92 g/day with a median value of 74 g/day. The energy expenditure was significantly correlated with age (*p* = 0.0001, r= 0.417) and body weight (*p* = 0.0001, r= 0.547). Similar correlations were obtained between the body weight for age percentile and the BMR value, where a significant positive correlation was obtained (*p* = 0.0006).

An increased BMR was significantly correlated with oxygen consumption (*p* < 0.0001, r = 0.928). However, active muscle mass increased the BMR value (*p* = 0.0001, r = 0.562), in contrast to the individuals with lower active mass (*p* > 0.05). The increases in daily energy demands and RQ were correlated with a significant increase in PAS (*p* = 0.0001, r = 0.373). However, the oxygen need was higher in individuals who had lower fat mass (*p* = 0.0002, r = −0.298) and an increased fat metabolism activity (*p* = 0.0001, r = 0.423).

### 3.3. Blood Samples and Anthropometric Analysis

Several parameters were significantly correlated with the participants’ body weight and body mass. Of them, the most important described the relationship between the total cholesterol, triglycerides, and body weight compared with body mass, as detailed in Table 2.

Total cholesterol, triglycerides and total proteins failed to correlate (*p* > 0.05) with both IL-6 and IL-8.

### 3.4. IL-6 and IL-8 and Anthropometric Changes

IL-6 was between 0.05 and 5.98 with a 1.4 pg/mL median value, whereas IL-8 was between 0.72 and 38.9, with a 7.09 pg/mL median value. IL-6 was significantly correlated with the skinfold sum, as confirmed through *p* = 0.0289, r = 0.187, which indirectly described a higher body mass (*p* < 0.05) and higher IL-6 values. Somewhat similarly, changes in the sum of the skinfold were significantly correlated with total proteins (*p* = 0.019, r = 0.200) and triglycerides (*p* = 0.015, r = 0.208). Both weight for age (*p* = 0.0001, r = 0.391) and height for age (*p* = 0.006, r = 0.218) were significantly correlated with the skinfold sum, while active body mass was negatively correlated (*p* = 0.0069, r = −0.373). In contrast, IL-8 did not correlate (*p* > 0.05), as detailed in Table 3.

### 3.5. Interleukins and Changes in Energy Needs

IL-6 did not correlate with total cholesterol and triglycerides values (*p* > 0.05), or weight for age and height for age percentiles (*p* > 0.05). However, through the BMR measurements, peak carbohydrate metabolism had changed significantly based upon the IL-6 value (*p* = 0.0158, r = 0.202) which was significantly correlated with the respiratory coefficient value (*p* = 0.0271, r = 0.185). The respiratory coefficient changed along with both total cholesterol (*p* = 0.013, r = 0.283) and triglycerides (*p* = 0.0006, r = 0.283) serum values, showing an indirect relationship with IL-6. Higher total cholesterol values were seen in individuals with higher triglycerides (*p* = 0.0003, r = 0.29) and higher body weight (0.0001, r = 0.376), but lower active mass (*p* = 0.0002, r = −0.306). However, total cholesterol was significantly correlated with lower oxygen consumption (*p* = 0.0019, r = −0.257), but with higher RQ values (*p* = 0.013, r = 0.208).

IL-8 failed to correlate with any of the anthropometric measurements (*p* < 0.05), but with daily energy needs. An increased IL-8 ratio was related to RQ value (*p* = 0.0211, r = 0.194) which therefore confirmed a negative correlation between IL-8 and the fat metabolism (*p* = 0.0042, r = −0.240). An increased IL-8 ratio was significantly correlated with lower age. Overall, RQ was significantly correlated with the main blood analyses: higher total cholesterol (*p* = 0.013, r = 0.208) and triglycerides (*p* = 0.0006, r = 0.283), as well as changes in creatinine value (*p* = 0.0105, r = −0.214). The same results were concluded over RQ measurements and body weight for age (*p* = 0.0089, r = 0.208), due to low fat metabolism activity (*p* = 0.0001, r = −0.825) over higher VCO_2_ ratios (*p* = 0.0009, r = 0.261), as further detailed in Table 4.

## 4. Discussion

In our research, the basal metabolic rate changed with both the skinfold sum and the fat mass percentage. Perhaps the most eloquent association was between the body mass and the serum blood samples. Some of these results illustrate serum values different from normal, with changes in body mass. However, IL-6 and IL-8 failed to correlate directly with the anthropometric measurements.

### 4.1. Anthropometric Changes and Energy Expenditure

Based on our results, the resting energy expenditure changes with body mass, energy intake, and with age. Under similar factors, changes in body mass occur more frequently in adult participants due to lower energy expenditure over positive changes in fat mass [47].

From the literature review, the energy expenditure components, such as the basal metabolic rate, physical activity, and food thermogenesis are not frequently detailed [50]. All these parameters can influence the measurements. There are still questions referring to differences in energy expenditure due to body mass changes [51]. Based on our outcomes, the main differences are related to the body mass. However, according to Westerterp K.R. et al. [52], the main changes in the energy expenditure are due to active tissue, whereas important differences are seen in females compared with male participants. Furthermore, in our study, a higher RQ value was mostly seen in participants with higher fat mass, while according to Piaggi P. et al. [53], changes in body mass increase energy expenditure and therefore carbohydrate metabolism by oxygen consumption, as seen in our paper. However, we must take into account the limitations of using RQ, which is influenced by the food quotient (FQ), the energy balance and the glycogen storage over a 24 h period, as seen in outcomes from Miles-Chan J.L. et al. [54].

Energy intake greatly influences the BMR and the changes in body mass. Based on our BMR measurement, a limited energy expenditure will not necessarily be associated with weight gain. According to Shook R.P. et al. [55], a higher resting metabolic rate and a lower respiratory coefficient may limit additional weight gain, unlike in our results, where we reported an increased respiratory coefficient and therefore an increased risk of weight gain. Resting energy expenditure changes with body weight and body mass, whereas an increased energy expenditure will usually increase the RQ, while lowering fat metabolism, as earlier seen in our paper. However, according to Suarez-Varela M.M. et al. [56], the energy intake mostly influences the anthropometric development. We cannot agree more, taking into account that on many occasions, participants with normal weight had low energy requirements. Moreover, energy intake was higher than the requirement in numerous cases in our study, which was the reason why the skinfold measurements were higher than normal in more than 70% of the participants, even though the body weight was normal. According to Gravio D.C. et al. [57], this may be the first change in the body mass, which can therefore increase the risk of obesity. This statement suggests information regarding the lack of changes in the inflammatory status, regardless of the body weight, as we will further discuss based on our results.

### 4.2. Total Proteins, Creatinine, Fat Profile, Energy Demands and Changes in Anthropometry

In our study, changes in blood serum were mostly related to the body mass. Even if the relationship between body weight, BMI and inactive body mass was previously determined [58], many other anthropometric changes have been seen in cases with related health risks [59]. For instance, the inflammatory status increases in adults with higher inactive mass, unlike our case. In our results, inactive mass (16.7% of the body mass) was related to the sum of the skinfolds (58.75 mm), which was correlated with both IL-6 and IL-8. Thus, indirectly, we can observe the relationship between body mass and inflammatory status. However, the extent to which the body mass causes changes in inflammatory status has not yet been defined, especially in young individuals. This hypothesis can also be confirmed by our research, which illustrated a lack of direct changes in inflammatory status, regardless of body weight.

Based on our results, the relationships between total cholesterol, triglycerides, total proteins and body weight increase the risk of several comorbidities. According to Ormazabal et al. [60], higher total cholesterol levels increase cardiovascular risk and insulin sensitivity. In addition, an increased cholesterol and triglycerides ratio, as well as changes in skinfolds, will significantly increase the risk of obesity [61]. The whole condition is quite similar to our outcome. We can therefore appreciate that changes in total cholesterol and triglycerides are usually seen with changes in body weight. In our paper, total proteins failed to change according to the body mass. Regarding the results from Madhuvannthi M. et al. [62], the serum proteins were considered normal, taking into account the study sample and the anthropometric results. However, total serum proteins decreased in underweight, in contrast to our sample.

In our paper, serum creatinine increased in participants with higher body weight, independent of the level of adiposity. According to Gerchman F. et al. [63] this association tends towards the relationship between body weight and the risk of kidney damage, similarly to how cholesterol increases the risk of cardiovascular events. However, various studies [64,65] suggest that blood pressure also increases with creatinine, while insulin sensitivity relates to a lower glomerular filtration rate (GFR). Further research is needed because other studies [66] state that the level of creatinine will not change with the level of adiposity but with the lean body mass, in contrast to our study results. Overall body mass changed serum blood parameters, and therefore increased the risk of other comorbidities.

### 4.3. Inflammatory Status: Anthropometric Changes and Differences in Energy Demands

In contrast with earlier published papers, our results suggest that age can influence IL responses. Chronic versus acute changes can probably have an important role in the response amplitude with body weight changes. According to Rea I.M. et al. [67], age-associated chronic diseases can increase pro-inflammatory cytokines. Based on our results, body weight failed to correlate with the inflammatory status of young individuals. However, the skinfolds were both related to IL-6 and IL-8 values, which may increase the relationship with the adipose mass. Skinfolds are often used to determine the levels of both lean and adipose tissue. The idea that IL-6 and IL-8 are mostly released from the adipose tissue may explain some of our results. However, the endocrine function of the adipocytes assures a bidirectional communication [68] with other tissues, especially during an inflammatory state. According to Surmi B.K. et al. [69], during the inflammatory state, the adipocyte tissue is infiltrated by macrophages, which can further lead to a local inflammation with IL-6 and IL-8 secretion.

The number of papers conducted on the inflammatory status in children is relatively small. In our research, IL levels failed to correlate with body mass. However, by comparing our results with other research, we further believe that children under the age of 13 do not develop a higher inflammatory status based on the body weight, somewhat similar to conclusions by Tam C.S. et al. [67,70,71]. This statement is based on other parameters, starting from total cholesterol, triglycerides, the BMR measurement, and the sum of skinfolds, which are all indirectly related to IL-6 and IL-8. The number of participants, the clear differentiation of the body weight category, and the detailed anthropometric analysis can help in the future to differentiate and identify early changes in inflammatory status.

## 5. Conclusions

Inflammatory status did not change with body weight. However, the adipose tissue influenced both IL-6 and IL-8 with regard to the skinfolds and energy expenditure. Most likely, the growth of adipose tissue attracts changes in serum parameters, by affecting the energy needs and by increasing the risk of associated pathologies. This would be the first stage in the development of inflammatory status through food intake and body weight.

## Figures and Tables

**Table 1:** Descriptive information regarding the anthropometric measurement results, illustrated as median value (minimum to maximum values).

Parameter	Median Value (Min to Max Values)
Body height	158 (124 to 166 cm)
Body weight	46.1 (22.7 to 78.5 kg)
Skinfold sum	58.75 (11 to 204 mm)
Fat mass	16.7 (6.6 to 39.3% of Fat mass)
Muscle mass	36 (31 to 45.5% of Muscle mass)
Weight for age	57 (0 to 115 %)
Height for age	67 (0 to 142%)
BMI for age	45.5 (1 to 105%)

Legend: cm—centimeters; kg—kilograms; mm—millimeters; %—percentage; Min—minimum; Max—maximum.

**Table 2:** Univariate analysis between anthropometric measures and cholesterol, triglycerides, and total proteins.

Anthropometric Parameters (Median Values, Min to Max)	Laboratory Parameters (Median Values, IQR)					
	Cholesterol (mg/dL) (144.7, 131.6 to 169.8)		Triglycerides (mg/dL) (49.46, 39.59 to 64.2)		Total Proteins (g/L) (69.13, 66.8 to 71.36)	
	*p*	r	*p*	r	*p*	r
Weight (kg): 46.1, 22.7 to 78.5	0.0001	0.376	0.9284	0.007	0.5022	0.057
Skinfold sum (mm): 58.75, 11 to 82.25	0.2366	0.102	0.0015	0.208	0.0192	0.200
BMI (kg/m^2^): 18.5, 13.2 to 25.3	0.0036	0.242	0.0749	0.156	0.8611	0.014
Weight for age (%): 57, 0 to 115	0.0013	0.208	0.8405	0.017	0.7252	−0.029
Height for age (%): 67, 0 to 142	0.0811	−0.146	0.9015	−0.010	0.5177	−0.054
Active body mass (%): 36, 32.1 to 45.5	0.0002	−0.306	0.2495	−0.097	0.3835	−0.073

Legend: Min—minimum; Max—maximum; mg/dL—milligrams per deciliter; g/L—grams per liter; *p*—probability level; r—Pearson product-moment correlation coefficient; BMI—body mass index; IQR—interquartile ranges.

**Table 3:** Univariate analysis between interleukins and anthropometric measurements.

Anthropometric Parameters (Median Values, Min to Max)	IL (Median Values, IQR)			
	IL-6 (pg/mL) (1.415, 0.75 to 2.18)		IL-8 (pg/mL) (7.09, 5.46 to 10.24)	
	*p*	r	*p*	r
Weight (kg): 46.1, 22.7 to 78.5	0.9085	0.009	0.0659	0.154
Skinfold sum (mm): 58.75, 11 to 82.25	0.0289	0.187	0.0767	0.150
BMI for age (%): 45.5, 1 to 105	0.2019	0.107	0.5168	0.056
Weight for age (%): 57, 0 to 115	0.9494	0.005	0.1992	−0.109
Height for age (%): 67, 0 to 142	0.2663	−0.093	0.7798	0.023
Active body mass (%): 36, 32.1 to 45.5	0.1921	−0.110	0.9121	−0.009

Legend: Min—minimum; Max—maximum; pg/mL—picograms per milliliter; *p*—probability level; r—Pearson product-moment correlation coefficient; BMI—body mass index; kg—kilograms; mm—millimeters; %—percentage; IQR—interquartile ranges.

**Table 4:** Statistical analysis between energy expenditure and interleukin test results.

Anthropometric Parameters (Median Values, Min to Max)	Laboratory Parameters (Median Values, IQR)									
	IL-6 (pg/mL) (1.415, 0.75 to 2.18)		IL-8 (pg/mL) (7.09, 5.46 to 10.24)		Cholesterol (mg/dL) (144.7, 131.6 to 169.8)		Triglycerides (mg/dL) (49.46, 39.59 to 64.2)		Total Proteins (g/L) (69.13, 66.8 to 71.36)	
	*p*	r	*p*	r	*p*	r	*p*	r	*p*	r
RQ: 46.1 (22.7 to 78.5)	0.027	0.185	0.021	0.194	0.013	0.208	0.006	0.283	0.186	−0.111
VO_2_ (mL): 58.75, (11 to 82.25)	0.999	−0.000	0.639	−0.039	0.001	−0.257	0.951	0.005	0.834	0.017
VCO_2_ (mL): 18.5, (13.2 to 25.3)	0.186	0.111	0.619	0.042	0.050	−0.164	0.402	0.070	0.835	0.017
FAT (g/day): 57, (35 to 115)	0.063	−0.156	0.004	−0.240	0.003	−0.299	0.0123	−0.209	0.074	0.156
CHO (g/day): 67, (0 to 142)	0.015	0.202	0.642	0.039	0.905	0.010	0.216	0.104	0.891	−0.011
BMR (kcal/day): 36, (32.1 to 45.5)	0.835	0.017	0.565	−0.049	0.006	−0.283	0.866	−0.014	0.620	−0.041

Legend: Min—minimum; Max—maximum; pg/mL—picograms per milliliter; *p*—probability level; r—Pearson product-moment correlation coefficient; RQ—respiratory quotient; VO_2_—oxygen consumption; VCO_2_—carbon dioxide production; FAT—lipid metabolism; CHO—carbohydrate metabolism; BMR—basal metabolic rate; IQR—interquartile ranges.
